# New Low-Cost Ceramic Microfiltration Membranes for Bacteria Removal

**DOI:** 10.3390/membranes12050490

**Published:** 2022-04-30

**Authors:** Olivier Mountoumnjou, Anthony Szymczyk, Emilia Enjema Lyonga Mbambyah, Dayirou Njoya, Antoine Elimbi

**Affiliations:** 1 Laboratory of Applied Inorganic Chemistry, Faculty of Sciences, University of Yaounde 1, Yaounde P.O. Box 812, Cameroon; mountoumnjouolivier@gmail.com (O.M.); dayirou2000@yahoo.fr (D.N.); aelimbi2002@yahoo.fr (A.E.); 2 Department of Microbiology, Faculty of Medecine and Biomedical Sciences, University of Yaounde 1, Yaounde P.O. Box 1364, Cameroon; emilialyo@yahoo.co.uk; 3 Centre for the Study and Control of Communicable Diseases, Faculty of Medecine and Biomedical Sciences, University of Yaounde 1, Yaounde P.O. Box 8445, Cameroon; 4 CNRS, ISCR (Institut des Sciences Chimiques de Rennes)—UMR 6226, University Rennes, F-35000 Rennes, France

**Keywords:** microfiltration, ceramic membranes, natural resources, *Escherichia coli*, *Staphylococcus aureus*

## Abstract

Safe water provision in low-income countries is constrained by limited financial resources, and the problem is worsened during natural disasters. Thus, there is a need to develop efficient low-cost technologies for point-of-use water treatment. This work reports on the development of new ceramic microfiltration membranes made from mixtures of inexpensive raw materials available locally (kaolin, bentonite and limestone) and their efficiency in rejecting bacteria such as *Escherichia coli* and *Staphylococcus aureus*. Thermogravimetric analysis, differential scanning calorimetry, Fourier-transform infrared spectroscopy, X-ray diffraction, mercury intrusion porosimetry, flexural strength and water uptake were used to characterize the raw materials and membranes. The addition of limestone in the membrane fabrication increased the pore size, the porosity and, thus, the permeability of the membranes but at the expense of the rejection performance. Among the different compositions studied, the membrane made of 83% kaolin, 10% bentonite and 7% limestone showed the best performance compromise with water permeability of 566 L·h^−1^·m^−2^·bar^−1^ and 100% rejection of both *Escherichia coli* and *Staphylococcus aureus*. These new low-cost microfiltration membranes are expected to have potential applications in water treatment and household applications.

## 1. Introduction

The lack of access to safe water is a challenge in many developing countries, especially in rural areas [1]. It is urgent to develop efficient and low-cost water-purification technologies to ensure drinking-water safety in these areas, because safe drinking water is essential to human health [2,3,4]. As reported by the World Health Organization (WHO), over 1.8 billion people lack access to safe drinking water around the world [5]. The exposure to water with poor quality can result in a variety of waterborne diseases such as diarrhea, cholera, dysentery, typhoid… [5,6,7]. It is estimated that approximately 1.7 million deaths per year worldwide are attributable to unsafe water, sanitation and hygiene [8]. Therefore, the primary goal of water-quality management from the health perspective is to ensure that consumers are not exposed to pathogens. Protection of water sources and appropriate treatment of water supplies have greatly reduced the incidence of these diseases in developed countries [9]. Access to water for populations is, therefore, a major challenge for the decades to come. Concerns associated with a growing drinking-water shortage and the use of physicochemical treatments associated with potential secondary pollution as well as excessive energy consumption, have generated a wave of technological innovations in the field of water filtration. Membrane separation processes, which are basic elements in chemical engineering, offer many advantages, since they are relatively energy efficient [10] (no phase change), clean (in the sense that they require few chemicals), modular and compact. Moreover, they can be integrated into conventional systems such as fermenters and reactors [11]. The significant improvements made in synthetic-membrane development over the past decades make membrane processes excellent candidates for addressing the issues outlined above. It is now commonly acknowledged that membrane processes can be considered as high-performance separation techniques in many industrial applications [12]. The interest in membrane separations has rapidly increased during the last 10–15 years [13,14] in various fields: chemistry, food, biotechnology, and wastewater treatment [15]. For instance, Tomczak and Gryta successfully applied ultrafiltation for the pretreatment of 1,3-propanodiol fermentation broths [16]. The same authors demonstrated the potentiality of capillary polypropylene membranes for the microfiltration of oily wastewater [17]; while Zielinska et al. showed that microfiltration and ultrafiltration membranes used in membrane bioreactor technology (in the place of a secondary clarifier) led to permeates that could be suitable for water reuse for irrigation purposes [18]. Ceramic membranes have several advantages over their organic counterparts, such as better thermal, chemical and mechanical resistances, bacteria resistance, high abrasion resistance, high permeability, long service life [19,20,21]. As pointed out by Tomczak et al., ceramic membranes can be efficiently cleaned under harsh environmental conditions without the risk of damaging membrane integrity [22]. According to Zielinska et al., ceramic membranes are also less prone to fouling than polymer ones, because bonding between foulants and ceramic membranes is weaker due to their hydrophilic surface properties [18]. However, these membranes are usually made from expensive metal oxides such as alumina, silica, zirconium dioxide and titanium dioxide [23], and the cost of ceramic membranes used in industrial applications is in the range 500–1000 $/m^2^ [24]. Alternatively, geomaterials are low-cost materials that need a lower sintering temperature than metal oxides. Indeed, the sintering temperature of metal oxides is about 1500 °C, while that of geomaterials is usually less than 1000–1200 °C [24,25]. Using cheaper raw materials is, then, recommended, to develop new ceramic membranes, especially for emerging countries where many environmental problems need to be solved at low cost. In this regard, as geomaterials are abundant in countries such as Cameroon, they can be used as alternatives to conventional ceramic membranes for water treatment. During the filtration process, substances are deposited onto the membrane surface, which reduces their permeation flux and is likely to modifiy their rejection performance. Membrane fouling is associated with the deposition and accumulation of organic (humic acids, proteins, carbohydrates, nano/microplastic…), inorganic (e.g., salts) and/or biological substances (micro-organisms) onto the membrane surface [20]. Fouling decreases process productivity and reduces membrane lifetime [26]. To ensure process durability and to maintain membrane performance, physical cleaning (water rinse, backwashing) and chemical cleaning (bleach, hydrogen peroxide, nitric acid, sodium hydroxide…) are required [21,26].

This work aimed at developing new lost-cost microfiltration membranes from kaolin, bentonite and limestone collected in several areas of Cameroon, and evaluating their efficiency in rejecting *Escherichia coli* and *Staphylococcus aureus* bacteria from contaminated water.

## 2. Materials and Methods

### 2.1. Raw Materials

Kaolinitic and bentonitic clays and limestone were collected from west, north-west and north regions of Cameroon, respectively. After crushing into small fragments, the samples were dried at 105 °C for 24 h, ground into powder in a ball mill, and passed through a 100 μm sieve. 

### 2.2. Membrane Preparation

Seven mixtures of kaolin, bentonite and limestone powders were considered for the membrane fabrication (see Table 1). The various mixtures of fine raw-material powders were shaped by uniaxial pressing to obtain rigid matrices. Two kinds of flat-sheet membranes were developed: (i) circular membranes of 47 mm diameter and 6 mm thickness and (ii) rectangular membranes of 82 mm length and 44 mm width (rectangular samples were used for flexural-strength measurements). For both geometries, four samples of each composition were made.

A certain mass of powder (25 g and 50 g for circular and rectangular samples, respectively) was humidified with a volume of distilled water (3 mL and 5 mL for circular and rectangular samples, respectively) in a mortar. After homogenization, the suspension was transferred into a steel mold and compacted with a hydraulic press at 30 MPa for 30 s.

After partial drying at room temperature for 24 h, the samples were baked at 105 °C for 24 h. The temperature was further increased up to 1150 °C with a heating rate of 2 °C/min and the samples were sintered at 1150 °C for 1 h.

### 2.3. Mechanical Properties 

The mechanical properties of the membranes were evaluated with a three-point bending method using an electro-hydraulic press (M&O, Type 11.50, N^o^ 21) at an average rate of 3 mm/min. The flexural strength was determined from the following expression [27]:

(1) $$\beta =\frac{3\;F\;l}{2\;b\cdot h^{2}}$$

where β is the flexural strength (MPa), F is the load at the fracture point (N), l is the span length (m), b is the sample width (m), and h is the sample thickness (m). 

For each membrane composition, the flexural strength reported hereafter is the average of measurements performed with four samples.

### 2.4. Water Uptake

The dry membranes were first weighed, then immersed in a beaker containing distilled water, boiled for 2 h and further left to cool for 24 h at room temperature. After cooling, the wet membranes were blotted with an absorbent paper and then weighed again. The water uptake (W_a_) was determined according to [28]:

(2) $$W_{a}=100\;\frac{\left(M_{w}\;–{\;M}_{d}\right)\;}{{\;M}_{d}}$$

where M_w_ is the mass of the wet membrane and M_d_ the mass of the dry membrane.

### 2.5. Mercury Intrusion Porosimetry (MIP)

The pore-size distribution was determined by MIP. The analysis was performed with a Quantachrome, AutoPore IV 9500 Micromeritics porosimeter. 

### 2.6. X-ray Diffraction (XRD)

XRD analysis was performed with powder (dried 100 µm fraction) by means of an Empyrean diffractometer (Malvern Panalytical Ltd., Malvern, UK) with Ni filter Cu Kα radiation (k = 1.5406 Å). The XRD patterns were recorded over the 2°–45° 2θ angular range using a scan step of 0.02° and a time step of 2 s. 

### 2.7. Attenuated Total Reflectance—Fourier-Transform InfraRed (ATR-FTIR) Spectroscopy

ATR-FTIR spectra were recorded with a Spectrum Two spectrometer (PerkinElmer, UK) equipped with a diamond crystal and operating in the wave-length range 400–4000 cm^−1^ with a resolution of 4 cm^−1^. Results shown in this work are the averages of seven scans. 

### 2.8. Chemical and Thermal Analyses 

Chemical analyses were carried out using emission spectrometry. Approximately 1 g of clay (100 µm powder) was molded in fused lithium borate (LiBO_2_) and dissolved in nitric acid. Inductively coupled plasma-atomic emission spectroscopy (ICP-AES) was used to determine major elements and inductively coupled plasma mass spectrometry (ICP-MS) was used for trace elements. Thermogravimetric analyses were carried out on ~45 mg of clay (100 µm fraction) using a TGA-DSC instrument (SETARAM) with a heating rate of 5 °C/min from ambient temperature to 1200 °C.

### 2.9. Membrane-Separation Performance 

Gravity-driven filtration was carried out with water collected from the Tam river (Bangourain, Cameroon). The water level above the membrane was kept equal to 13 cm during the entire filtration experiment. The membrane hydraulic permeability (L_p_) was determined according to [29]:

(3) $$L_{p}=\frac{V}{A\cdot \Delta t\cdot \Delta P}$$

where V is the volume of water filtered, A is the membrane surface area, Δt is the sampling time and ΔP is the transmembrane pressure difference. 

Two common pollutants in drinking water are the model bacteria, *Escherichia coli*, a rod-shaped bacterium with length of 2000–5000 nm and width of 400–600 nm; and *Staphylococcus aureus*, with a spherical size of about 1000 nm [30]. They were selected to model Gram-negative and Gram-positive bacteria, respectively. Additionally, they are often used as indicators of pathogenic bacteria [31].

The bacteria were first cultured, i.e., 15 g of Eosin Methylene Blue (EMB) agar culture medium (ATCC 25922 *E. coli*) and 33.6 g of Chapman culture medium (ATCC 25923 *S. aureus*) were introduced into two separate 1000 mL beakers and then 400 mL of distilled water was added to the beaker containing EMB and 300 mL of distilled water were added to that containing Chapman. Each beaker and its content was heated until boiling to homogenize the medium. After cooling down to room temperature, the two solutions were sterilized in an autoclave at 121 °C for 15 min and further poured (20 mL) into Petri dishes. After cooling, the agar plates were controlled for fertility, sterility and specificity. The plates were then inoculated at 37 °C for 24 h; EMB plates with *E. coli* (ATCC 25922) and Chapman agar plates with *S. aureus* (ATCC 25923). The colonies were confirmed by standard microbiological methods. A 10^5^ CFU/mL bacteria concentration in 20 mL of sterile distilled water was prepared. The mixtures were then vortexed sequentially before use. The permeate was inoculated into the corresponding medium, which were placed in an incubator for 24 h at 37 °C for the growth of potential bacteria passed through the membranes. 

The *E. coli* and *S. aureus* concentrations in the permeate were determined from the plaque-forming-unit method [32]. Each colony corresponding to a strain of bacteria, we counted in the Petri dishes the number of colony of bacteria that we divide by the volume of the inoculum (20 mL) to obtain the concentration of the bacteria in the permeate [33]. The rejection rate^®^ was determined from [34]:

(4) $$R=100\left(1-\frac{C_{p}}{C_{f}}\right)$$

where, C_f_ and C_p_ are the feed and permeate concentrations (CFU/mL), respectively. 

All experiments were duplicated.

## 3. Results and Discussion

### 3.1. Characterization of the Raw Materials

The formation of characteristic mineral phases and microstructures is impacted by the mineral content of the raw material, which depends on local geological sources [35]. The chemical composition of the various raw materials used for membrane synthesis is given in Table 2.

SiO_2_ and Al_2_O_3_, which are known to provide good mechanical properties [36], were the main oxides in both kaolin and bentonite. Bentonite was found to contain much more SiO_2_ (67.52%) than Al_2_O_3_ (15.08%), whereas kaolin contained closer amounts of SiO_2_ and Al_2_O_3_ (48.5 and 32.24%, respectively). Kaolin had a much lower Fe_2_O_3_ content (1.51%) compared to bentonite (5.09%). The amount of oxides such as TiO_2_, CaO and K_2_O, which can contribute to decreasing the melting temperature and lead to the appearance of a vitreous phase after cooling, was 2.36, 0.05, and 1.16%, respectively, in kaolin, while bentonite had 0.26, 0.70 and 0.75% of TiO_2_, CaO and Na_2_O, respectively. Limestone had 56.04% of CaO. Both kaolin and bentonite contained a very low amount of magnesium and calcium oxide. The relatively high TiO_2_ content in kaolin (2.36%) suggests the presence of minerals such as rutile and/or anatase [37,38].

The limited loss on ignition measured with kaolin and bentonite (13.39% and 9.06%, respectively) is associated with the small amount of carbonates and organic matter in the raw materials [39].

**Figure 1:**
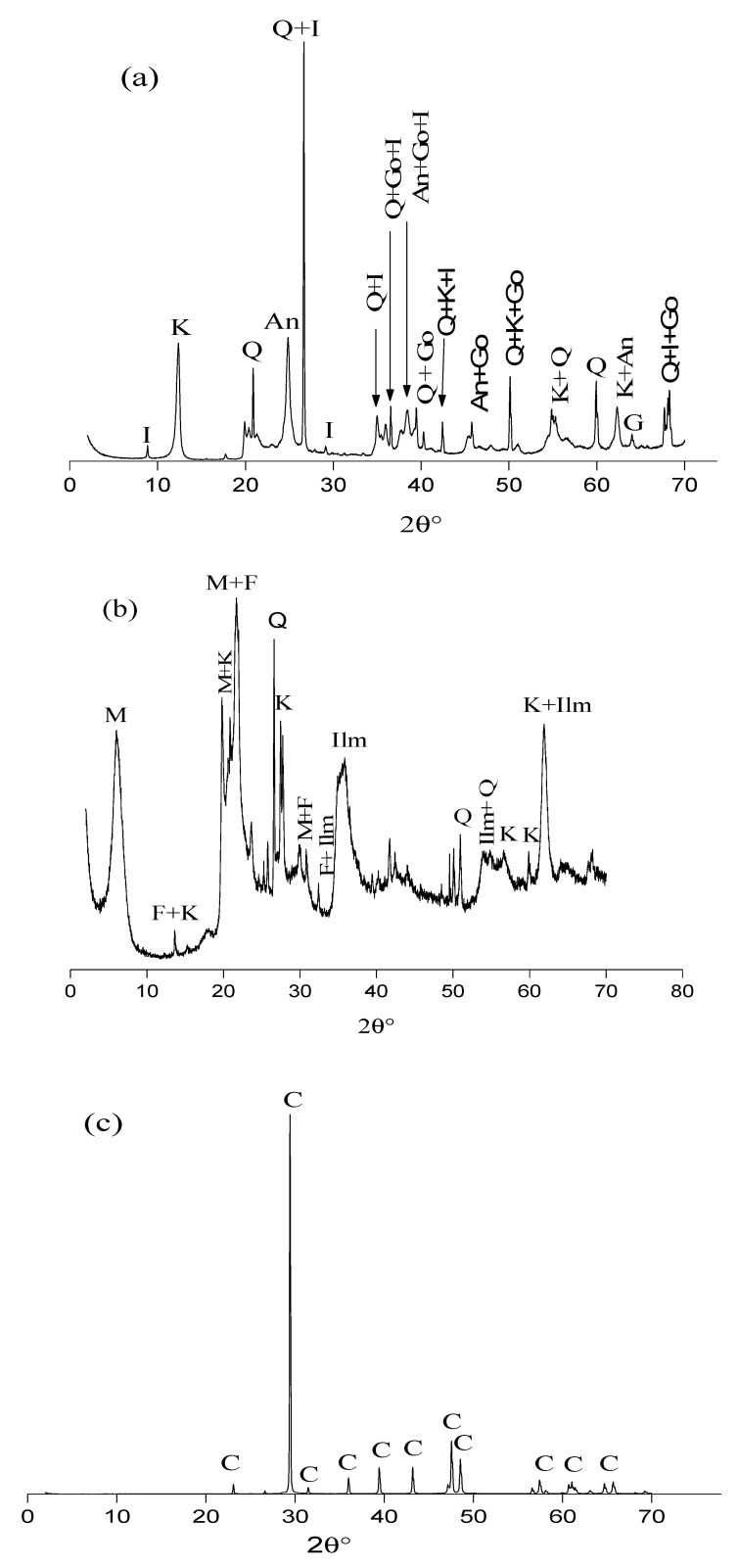
XRD diffraction patterns of the raw materials (**a**) kaolin, (**b**) bentonite, and (**c**) limestone. M: montmorillonite; I: illite; Q: quartz; C: calcite; K: kaolinite; F: feldspath; Ilm: ilmenite; An: anatase; Go: goethite.

Figure 1 shows the XRD patterns of the three raw materials. The phases responsible for the peaks observed on the various patterns were identified from the Powder Diffraction File (PDF) standards from the International Centre for Diffraction Data [40].

Both kaolin and bentonite contained quartz and kaolinite (Figure 1a,b), which are known to provide low plasticity, high refractory properties and hydrophilic properties [24]. After a heat treatment from 800 to 1100 °C, these minerals are transformed into a liquid phase playing the role of a linker between particles [41,42], which leads to better mechanical properties [43]. Kaolin also contained illite, anatase and goethite. The XRD pattern of bentonite (Figure 1b) shows it contained several minerals such as montmorillonite, which has swelling properties and whose fusion during heat treatment allows particle consolidation [44,45]. However, an excess of this mineral could also promote an abundance of the vitreous phase, which might decrease the membrane mechanical resistance [46]. 

The XRD pattern of limestone (Figure 1c) shows that it was pure, containing only calcite. The latter is likely to act as a pore-forming agent due to the release of carbon dioxide during heat treatment. 

Kaolinite was found to be the main mineral in kaolin (63%), followed by quartz (26%) and illite (8%), while the main minerals in bentonite were montmorillonite (54%), quartz (25%) and feldspar (18%) (Table 3). The large amount of montmorillonite in bentonite reinforces the role of plasticizer it can play during heat treatment. It is worth noting that kaolin also contained illite, which is expected to increase the plastic behavior of the clay mixture [47,48].

Limestone used as raw material in this study was 99.99% pure, i.e., it contained calcite (CaCO_3_) at 99.99%. 

Thermal analysis aimed at identifying the temperature zones where membrane mass losses and transformations occurred. Heat treatment may result in a loss of H_2_O, CO_2_ and/or organic matter, as well as a number of transformations of the various minerals present in the raw materials. 

**Figure 2:**
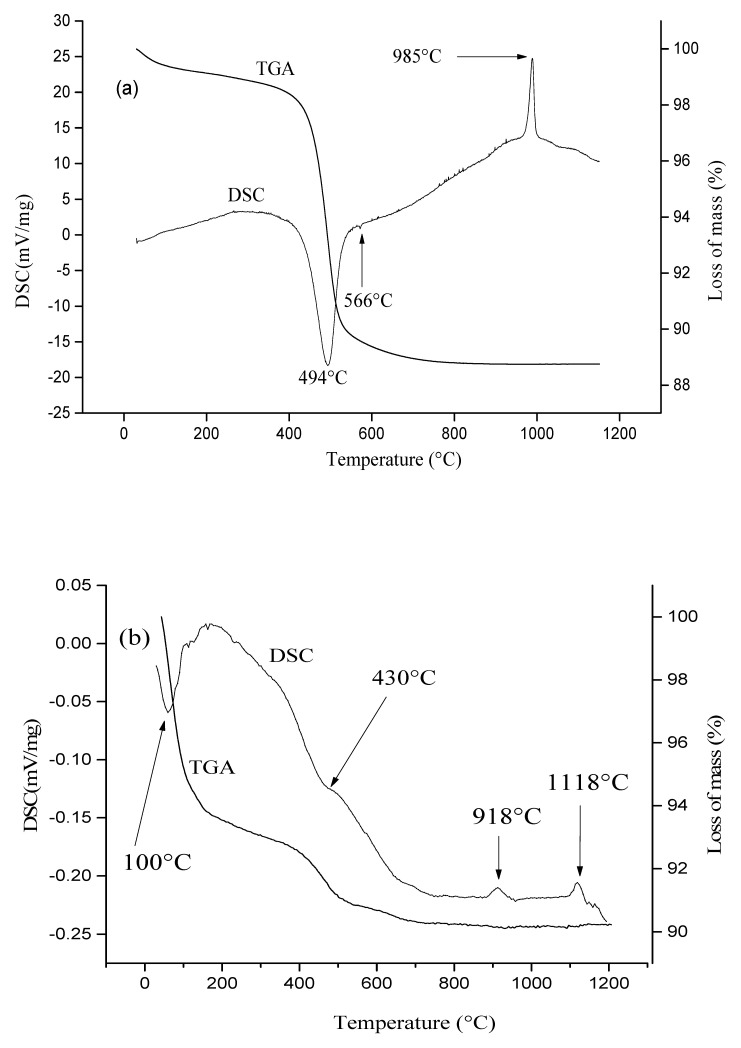

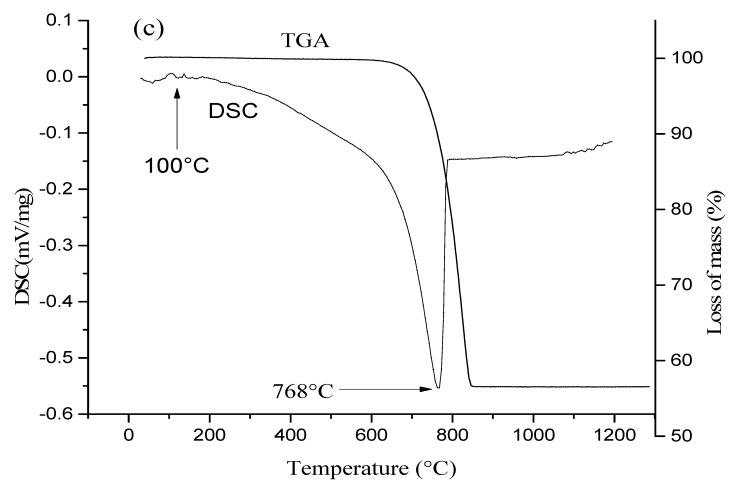
TGA–DSC curves of the various raw materials: (**a**) kaolin, (**b**) bentonite, and (**c**) limestone.

The kaolin thermogram (Figure 2a) shows a strong endothermic peak at 494 °C, which is associated with kaolinite dehydroxylation (Al_2_Si_2_O_5_(OH)_4_ → Al_2_Si_2_O_7_ + 2H_2_O). A weak endothermic peak was also detected at 566 °C, which corresponds to the polymorphic change from α-quartz to β-quartz. An exothermic event associated with the structural reorganization of metakaolinite into spinel or mullite can be seen at 985 °C. 

The bentonite thermogram (Figure 2b) shows (i) an endothermic peak around 100 °C, which is associated with a loss of moisture and/or adsorbed water, (ii) a shoulder peak around 430 °C, assigned to kaolinite dehydroxylation, and (iii) two exothermic events at 918 °C and 1118 °C, which are associated with the structural reorganization of metakaolinite and the release of mullite during demixing, respectively [49,50].

The differential scanning calorimetry (DSC) curve of limestone (Figure 2c) shows an intense endothermic peak at 768 °C that reflects the decomposition of calcite CaCO_3_ according to [51]: CaCO_3_ $\overset{700–950\;{}^{\circ}C}{\to }$ CaO + CO_2_.

**Figure 3:**
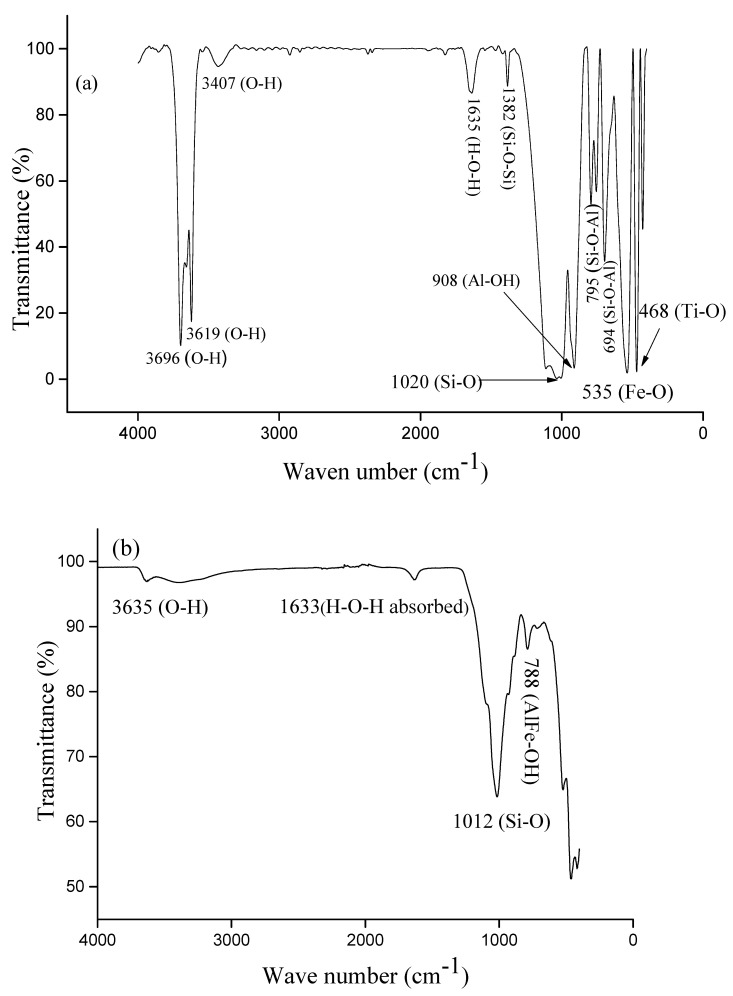

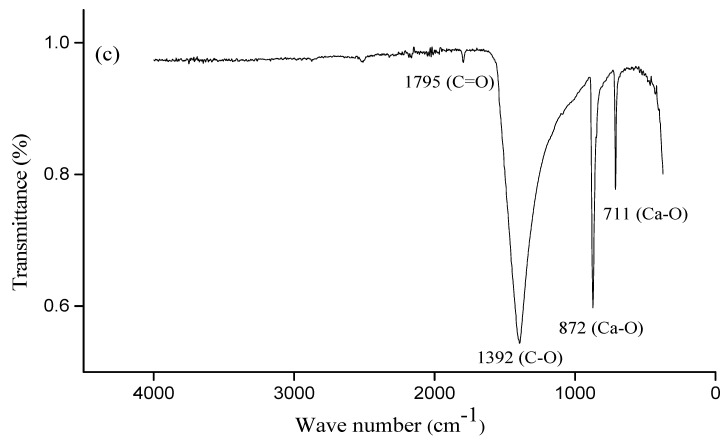
FTIR spectra of kaolin (**a**), bentonite (**b**) and limestone (**c**).

The ATR-FTIR spectrum of kaolin (Figure 3a) shows bands at 3407, 3619 and 3696 cm^−1^ that are associated with O-H stretching (AlOH of smectite [52] and hydroxyl groups of kaolinite). This figure also shows a wide band of water around 1635 cm^−1^ that corresponds to the symmetrical stretching of the H-O-H bond [53,54] (water absorbed onto the material) while the band at 1382 cm^−1^ is associated with the Si-O-Si bond [55]. The band observed at 908 cm^−1^ corresponds to the Al-OH bond, whereas those observed at 694 and 795 cm^−1^ are attributed to the Si-O-Al bond of montmorillonite [56,57]. The bands at 468, 535, and 1020 cm^−1^ correspond to Ti-O, Fe-O (present in Goethite), and Si-O bonds, respectively [58].

The ATR-FTIR spectrum of bentonite is shown in Figure 3b. It shows the bands at 3635 and 1633 cm^−1^ corresponding to O-H groups and H-O-H bonds respectively. The band at 1012 cm^−1^ is attributed to the Si-O stretching band of both di-and tri-octahedral smectites and montmorillonite [59]. The band observed at 788 cm^−1^ is assigned to the deformation δOH of AlFe-OH [60,61].

Figure 3c shows the ATR-FTIR spectrum of limestone. The band at 1795 cm^−1^ is attributed to the vibration of the CO double bond and the band at 1392 cm^−1^ is associated with the vibration of the CO single bond. Bands observed at 872 cm^−1^ and 711 cm^−1^ are attributed to the vibration of the Ca-O bond [62,63].

### 3.2. Membrane Characterization

**Figure 4:**
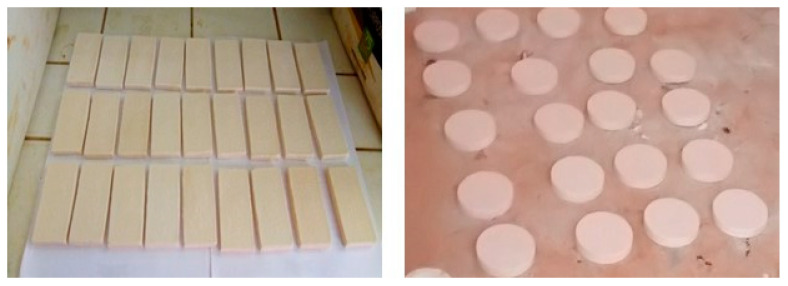
Physical aspect of membranes after sintering at 1150 °C.

After sintering, the membranes exhibited a nice physical aspect (see Figure 4) without cracks and defects and a pinkish white coloration (10R8/2) [64] due to the joint presence of alumina and iron oxide in the raw materials and calcium oxide resulting from the decomposition of calcite (CaCO_3_) during sintering [42,43,44,45,46,47,48,49,50,51,52,53,54]. 

**Figure 5:**
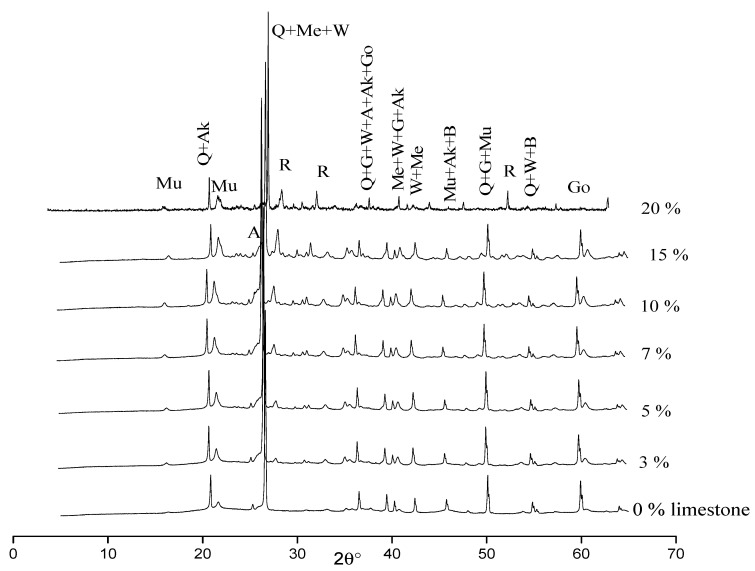
Membrane XRD patterns. Q: quartz, Mu: mullite, R: rutile, G: gehlenite, Ak: akermanite, Me: merwinite, W: wollastonite, A: anorthite, B: biotite, Go: goethite.

Figure 5 shows the XRD patterns of the various membranes. The same inorganic compounds were found in the various membranes after sintering except for the membrane containing 20% of limestone for which the peak of goethite was not detected. Calcium carbonate or calcium oxide presents in the raw materials (or formed by the thermal decomposition of calcium carbonate) reacted with silica and metakaolinite to give anorthite (CaAl_2_Si_2_O_8_), gehlenite (Ca_2_Al_2_SiO_7_), wollastonite (CaSiO_3_) and mullite (Al_4.5_Si_1.5_O_9.75_), according to the following reactions [47,48,49,50,51]:

$$3{\mathrm{SiO}}_{2}\cdot {\mathrm{Al}}_{2}O_{3}+6\mathrm{CaO}\;\overset{T\;>\;920\;{}^{\circ}C}{\to }\;3{\mathrm{Ca}}_{2}{\mathrm{Al}}_{2}{\mathrm{SiO}}_{7}\;(\mathrm{gehlenite})$$

$$3{\mathrm{SiO}}_{2}\cdot {\mathrm{Al}}_{2}O_{3}+6\mathrm{CaO}\;\overset{T\;>\;920\;{}^{\circ}C}{\to }\;3{\mathrm{Ca}}_{2}{\mathrm{Al}}_{2}{\mathrm{SiO}}_{7}\;(\mathrm{gehlenite})$$

$${\mathrm{CaCO}}_{3}+{\mathrm{SiO}}_{2}\;\overset{T\;>\;920\;{}^{\circ}C}{\to }\;{\mathrm{CaSiO}}_{3}\;(\mathrm{wollastonite})+{\mathrm{CO}}_{2}$$

$${\mathrm{Ca}}_{2}{\mathrm{Al}}_{2}{\mathrm{SiO}}_{7}+3{\mathrm{SiO}}_{2}+{\mathrm{Al}}_{2}O_{3}\;\overset{T\;>\;920\;{}^{\circ}C}{\to }\;2{\mathrm{CaAl}}_{2}{\mathrm{Si}}_{2}O_{8}\;(\mathrm{Anorthite})$$

$${\mathrm{Al}}_{2}{\mathrm{Si}}_{2}O_{5}(\mathrm{OH}{)}_{4}\;\overset{T\;>\;550\;{}^{\circ}C}{\to }\;{\mathrm{Al}}_{2}{\mathrm{Si}}_{2}O5_{\;}(\mathrm{Metakaolinite})\;\overset{T\;>\;950\;{}^{\circ}C}{\to }\;{\mathrm{Al}}_{4.5}{\mathrm{Si}}_{1.5}O_{9.75}\;(\mathrm{Mullite})+{\mathrm{SiO}}_{2}$$

$$((\mathrm{Na},\mathrm{Ca}{)}_{0.3}(\mathrm{Al},\mathrm{Mg}{)}_{2}{\mathrm{Si}}_{4}O_{10}(\mathrm{OH}{)}_{2}\cdot n(H_{2}O))\mathrm{Bentonite}+\mathrm{CaO}\;\overset{T\;>\;550\;{}^{\circ}C}{\to }\;\mathrm{Vitreous}\;\mathrm{liquid}\;\mathrm{phase}\;\overset{T\;>\;850\;{}^{\circ}C}{\to }\;\mathrm{Anorthite}$$

The formation of these minerals is consistent with the presence of oxides such as silica (SiO_2_), calcium oxide (CaO), aluminum oxide (Al_2_O_3_), iron oxide (Fe_2_O_3_) and magnesium oxide (MgO). At 700 °C, CaCO_3_ begins to decompose, yielding CaO with release of CO_2_ from the fired body and almost disappearing calcite at the maximum firing temperature (1150 °C). The intensity of rutile and mullite peaks was found to increase with increasing limestone content. All the membranes contained gehlenite, akermanite, merwinite and wollastonite, which were formed during heat treatment. Anorthite, goethite and biotite were not detected in the membrane prepared with 20% of limestone, possibly due to reactions with other minerals present in the raw materials. 

**Figure 6:**
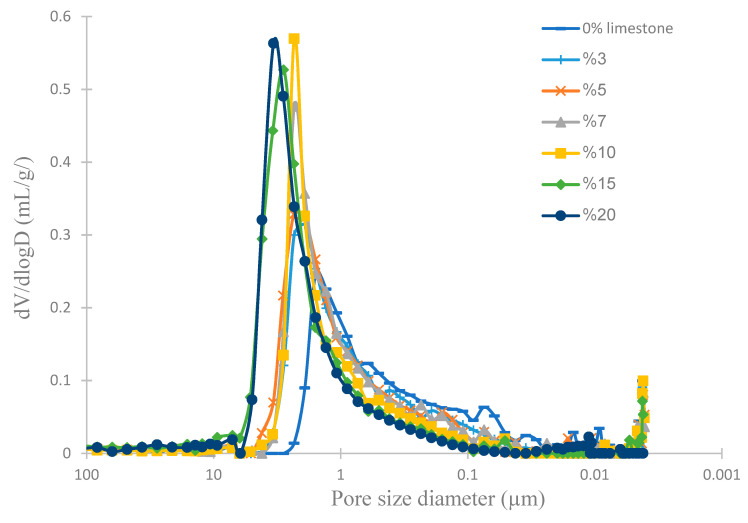
Membrane pore-size distributions.

MIP was used to determine the pore-size distribution of the various membranes (Figure 6). By increasing the limestone content, the pore-size distribution shifted towards larger pore sizes with the following average pore diameters (Table 4): 1.5 μm (0% limestone), 2.0 μm (3% limestone), 2.3 μm (5% limestone), 2.3 μm (7% limestone), 2.7 μm (10% limestone), 2.8 μm (15% limestone) and 3.4 μm (20% limestone). The increase in pore size with increasing limestone content is due to the increased release of carbon dioxide during sintering. These results highlight the role of limestone as a pore-forming agent. 

The increase in the pore size was found to be correlated with the increase in the membrane porosity and water uptake (Table 4); from 36% (0% limestone) to 44% (20% limestone) for the porosity and from 15.0% (0% limestone) to 18.4% (20% limestone) for the water uptake.

The addition of limestone was found to decrease the membrane flexural strength (Table 4) from 4.48 MPa for the membrane synthesized without limestone down to 0.26 MPa for the membrane with the highest content of limestone. These results can be explained by the increase in the membrane porosity, which makes the membranes less resistant to bending. It can be stressed that the flexural strengths of our membranes are in the range of reported values in the literature. For example, Mouafon et al. [65] obtained flexural strength between 1.07 and 7.79 MPa using kaolin, cassava starch and bovine bone ash as raw materials; while Iaich et al. [66] reached 1.98 MPa using clay powder and organic additives (polyvinyl alcohol). Nandi et al. [67] obtained a flexural strength of between 3 and 8 MPa using kaolin and other suitable low-cost materials such as quartz, sodium carbonate, calcium carbonate, boric acid and sodium metasilicate. 

### 3.3. Membrane Separation Performance

**Figure 7:**
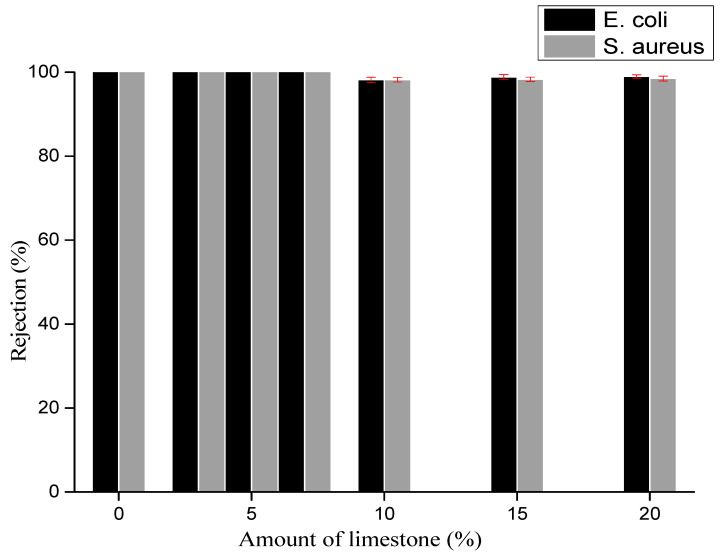
Bacteria rejection by the various membranes.

Figure 7 shows the rejection of bacteria by the various membranes after gravity-driven filtration carried out with a bacteria concentration of 10^5^ CFU/mL. The membranes prepared with less than 10% of limestone exhibited total rejection of both *E. coli* and *S. aureus*, which results from the membrane structure but also from the low driving force used in the experiments (gravity filtration). Indeed, Biron et al. observed a decreasing *E. coli* rejection from 98% down to 66% when increasing the transmembrane pressure applied through mullite ceramic membranes from 50 to 200 kPa [68]. 

The membranes synthesized with 10% or more of limestone had lower rejection performance, in the range 98–99%, *S. aureus* being less rejected compared to *E. coli*. The lower *S. aureus* rejection might result from the difference in the structure of the two bacteria. Indeed, Gram-positive and Gram-negative bacteria have different membrane structures, the most distinctive difference lying in the thickness of the peptidoglycan layer [69]. The peptidoglycan layer of the cell walls of Gram-positive bacteria (about 20–80 nm) is usually thicker than that of Gram-negative bacteria (about 7–8 nm) [70]. Moreover, *S. aureus* is less-negatively charged than *E. coli*, which may also contribute to its lower rejection [71]. 

The membranes developed in this work from natural raw materials therefore exhibit promising performance compared with the existing literature. For instance, Kamgang-Syapnjeu et al. reported a 90% rejection of *E. coli* with membranes elaborated from clays, coconut husks and eggshells [33]. Chaukura et al. obtained total rejection of *E. coli* with clay/sawdust composite membranes impregnated with silver nanoparticles [7]. He et al. investigated the removal of *E. coli* using clay/rice-husk filters decorated by TiO_2_ nanoparticles [1]. They achieved almost total *E. coli* rejection under UV radiation (and 99.4% rejection under dark conditions, i.e., when the photocatalytic activity of TiO_2_ was not activated). Zhu et al. also reported a complete removal of both *E. coli* and *S. aureus* by a mullite/carbon nanotube composite membrane [29]. However, the above-mentioned performance all required the addition of nanomaterials, which would undoubtedly increase the overall cost of the membrane.

Permeability experiments were carried out with water collected from the Tam river (Bangourain, west region of Cameroon). Membrane permeability depends on the membrane chemistry, porosity, pore size and thickness [72]. It was found to increase with the amount of limestone used for membrane preparation (Table 5), which correlates with the increase in the pore size and porosity revealed by MIP (Table 4). Interestingly, up to 7% of limestone, it was possible to significantly increase the membrane permeability (from 179 to 566 L·h^−1^·m^−2^·bar^−1^) while maintaining total bacteria rejection. For higher amounts of limestone, the membrane permeability still increased (up to 755 L·h^−1^·m^−2^·bar^−1^) but the rejection dropped due to the well-known rejection–permeability trade off [73].

From the above results, 83% kaolin, 10% bentonite and 7% limestone appear as the best composition to obtain low-cost ceramic membranes suitable for bacteria removal from contaminated waters. 

## 4. Conclusions

New low-cost microfiltration membranes have been developed from Cameroonian natural raw materials. Defect-free membranes made from kaolinitic clay, bentonite clay (10%) and limestone (from 0 up to 20%) were obtained after sintering at 1150 °C. Limestone played the role of pore former, as revealed by mercury intrusion porosimetry, which showed an increase in both the average pore diameter, from 1.5 to 3.4 µm, and porosity, from 36 to 44%, as the amount of limestone was varied from 0 to 20%. The increase in porosity was at the expense of the flexural strength of the membranes.

The efficiency of these low-cost membranes for the rejection of bacteria (*E.coli* and *S. aureus*) was evaluated by gravity-driven filtration. Membranes containing up to 7% limestone exhibited total rejection of both bacteria; while membranes containing more limestone performed worse with *E.coli* and *S. aureus* rejections of 99% and 98%, respectively.

The membrane permeability measured with local river water was found to increase with the amount of limestone used in membrane preparation, ranging from 179 L·h^−1^·m^−2^·bar^−1^ for the membrane without limestone to 755 L·h^−1^·m^−2^·bar^−1^ for the membrane elaborated with the highest amount of limestone (20%). The membrane elaborated from 83% kaolin, 10% bentonite and 7% limestone showed the most promising performance with a permeability of 566 L·h^−1^·m^−2^·bar^−1^, associated with a total rejection of *E.coli* and *S. aureus*.

## Figures and Tables

**Table 1:** Composition of the various membranes.

Kaolin (%)	90	87	85	83	80	75	70
Bentonite (%)	10	10	10	10	10	10	10
Limestone (%)	0	3	5	7	10	15	20

**Table 2:** Various oxides contained in the raw materials.

Sample	Content (%)											
	SiO_2_	Al_2_O_3_	Fe_2_O_3_	MnO	MgO	CaO	Na_2_O	K_2_O	TiO_2_	P_2_O_5_	L.O.I.	Total
Kaolin	48.50	32.24	1.51	-	0.28	0.05	-	1.16	2.36	0.19	13.69	99.98
Bentonite	67.52	15.08	5.09	0.01	0.28	0.70	0.75	1.25	0.26	-	9.06	100
Limestone	CaO (56.04)											

L.O.I = Loss on ignition.

**Table 3:** Mineralogical composition of the kaolin, bentonite and limestone.

Minerals (%) Sample	Kaolinite	Montmorillonite	Illite	Feldspar (Albite)	Anatase	Quartz	Ilmenite	Goethite
Kaolin	63	-	8	-	1	26	-	1
Bentonite	2	54	-	18	-	25	1	-
Limestone	Calcite (99.99)							

**Table 4:** Porosity, pore size, flexural strength and water uptake of the various membranes.

Limestone (%)	Porosity (%)	Average Pore Size (µm)	Flexural Strength (MPa)	Water Uptake (%)
0	36	1.5	4.48	15.0
3	38	2.0	4.10	15.6
5	39	2.3	3.78	16.0
7	40	2.3	1.96	16.0
10	42	2.7	1.17	16.5
15	43	2.8	0.39	17.0
20	44	3.4	0.26	18.4

**Table 5:** Membrane water permeabilities.

Limestone (%)	0	3	5	7	10	15	20
Water permeability (L·h^−1^·m^−2^·bar^−1^)	179 ± 15	357 ± 14	513 ± 18	566 ± 18	577 ± 7	579 ± 8	755 ± 17

## Data Availability

Data is contained within the article.
